# Interactions of Ascorbic Acid, 5-Caffeoylquinic Acid, and Quercetin-3-Rutinoside in the Presence and Absence of Iron during Thermal Processing and the Influence on Antioxidant Activity

**DOI:** 10.3390/molecules26247698

**Published:** 2021-12-20

**Authors:** Layla Engelhardt, Tobias Pöhnl, Susanne Neugart

**Affiliations:** Department of Crop Science, Division of Quality and Sensory of Plant Products, Georg-August-University Göttingen, Carl-Sprengel-Weg 1, 37075 Goettingen, Germany; tobias.poehnl@uni-goettingen.de (T.P.); susanne.neugart@uni-goettingen.de (S.N.)

**Keywords:** ABTS, antagonism, ascorbic acid, bioactive compounds, 5-caffeoylquinic acid, chelating complexes, ferrous iron, ferric iron, HPLC, quercetin-3-rutinoside synergism, TPC

## Abstract

Bioactive compounds in fruit and vegetables influence each other’s antioxidant activity. Pure standards, and mixtures of the common plant compounds, namely ascorbic acid, 5-caffeoylquinic acid, and quercetin-3-rutinoside (sum 0.3 mM), in the presence and absence of iron, were analyzed pre- and post-thermal processing in an aqueous solution. Antioxidant activity was measured by total phenolic content (TPC), 1,1-diphenyl-2-picrylhydrazyl (DPPH), and 2,2′-azino-bis (3-ethylbenzothiazoline-6-sulfonic acid) (TEAC) radical-scavenging assays. Ionic ferrous iron (Fe^2+^) and ferric iron (Fe^3+^) were measured photometrically. For qualification and quantification of reaction products, HPLC was used. Results showed that thermal processing does not necessarily lead to a decreased antioxidant activity, even if the compound concentrations decreased, as then degradation products themselves have an antioxidant activity. In all used antioxidant assays the 2:1 ratio of ascorbic acid and 5-caffeoylquinic acid in the presence of iron had strong synergistic effects, while the 1:2 ratio had strong antagonistic effects. The pro-oxidant iron positively influenced the antioxidant activity in combination with the used antioxidants, while ferrous iron itself interacted with common in vitro assays for total antioxidant activity. These results indicate that the antioxidant activity of compounds is influenced by factors such as interaction with other molecules, temperature, and the minerals present.

## 1. Introduction

In the human diet, many different plant-based products are consumed throughout the day, even within the same meal. Fruit and vegetables are associated with various health benefits, due to their variety of bioactive compounds, i.e., vitamins, minerals, and secondary plant metabolites [1]. In comparison to vitamins and minerals, secondary plant metabolites, such as phenolics, are not regarded as essential for human health by today’s knowledge [2]. However, many secondary plant metabolites are potent antioxidants, which help to protect biological systems from reactive oxygen species (ROS) and reactive nitrogen species (RNS) [3]. These bioactive compounds can interact with each other before consumption, resulting in numerous subsequent reaction products or complexes. Consequently, their contribution to consumers’ health and their antioxidant activity (AOA) may be altered [4] or lost before food intake. Different molecular structures of compounds and their diverse combinations can lead to additional, synergistic, or even antagonistic effects through interactions. Effects of interactions between plant components on their radical-scavenging ability are not fully understood yet, especially because of the many different oxidation stages and their ability to build complexes. A major bioactive compound, found in numerous plant species, is 5-caffeoylquinic acid (chlorogenic acid), a hydroxycinnamic acid ester of caffeic acid and quinic acid. It can form ferric iron complexes, which are generally associated with a reduced absorption of non-heme iron in humans [5,6]. Iron, as a component of hemoglobin, is an essential mineral for the human body, while an excess of iron can cause oxidative stress in cells [7]. Interactions with other nutrients can increase or decrease the absorption of iron, for example, by forming complexes. Furthermore, iron has the capacity to accept and donate electrons readily.

Another interesting bioactive compound in plants is quercetin-3-rutinoside (rutin), a flavonoid glycoside, which is mainly synthetized by plants to protect themselves from UV radiation [8,9]. It has a low aqueous solubility [10] and cannot enter human blood circulation directly, so its bioactive activity does not begin until six to nine hours after consumption, when it passes through the gut microbiome. In food supplementals, quercetin-3-rutinoside is often combined with ascorbic acid [11]. Ascorbic acid is an essential vitamin to the human body and needed in many metabolic processes. It is also a powerful water-soluble antioxidant, but can also act as a pro-oxidant, which is due to its ability to reduce ferric iron via chelate complex formation, followed by the transformation of ferrous iron and an ascorbic radical [12]. Increased absorption in the human body of the bioactive compounds quercetin-3-rutinoside, 5-caffeoylquinic acid, or iron was found by adding ascorbic acid [12], while 5-caffeoylquinic acid reduced the iron absorption by complexation [6]. To predict the health benefits of different products, in regard to bioactive compounds, in vitro antioxidant assays are commonly used. This prediction of health effects is limited due to variable bioavailability of compounds, metabolization, and different antioxidant mechanisms [13]. However, these assays are still useful to test for bioactive compounds, such as polyphenols, or, as in this study, to assess the AOA of single compounds and their interactions. Fruit and vegetables contain a diverse set of bioactive compounds, which can form complexes with other compounds and proteins that can interact with them. These interactions can influence the behavior of the compounds and the resulting AOA. However, this work is intended to contribute to the knowledge of how bioactive compounds interact with each other and their impact on the AOA. The novelty of this study is the interactions of common phenolic compounds, namly ascorbic acid, 5-caffeoylquinic acid, quercetin-3-rutinoside, with each other and the mineral iron pre- and post-thermal processing. Prior this study, these factors were evaluated separately, not in combination. The focus was set on the AOA and their ability to build complexes. The hypotheses were (1) thermal processing influences the AOA negatively due to degradation of the antioxidants, (2) mixtures in different ratios of ascorbic acid, 5-caffeoylquinic acid, and quercetin-3-rutinoside lead to a synergistic effect regarding the AOA, and (3) addition of the mineral and pro-oxidant iron will decrease antioxidant activity of the antioxidants alone or in mixtures.

## 2. Results

### 2.1. Influence of Thermal Processing on Antioxidant Activity of Ascorbic Acid, 5-Caffeoylquinic Acid, and Quercetin-3-Rutinoside Standards and the Mineral Iron

**Figure 1:**
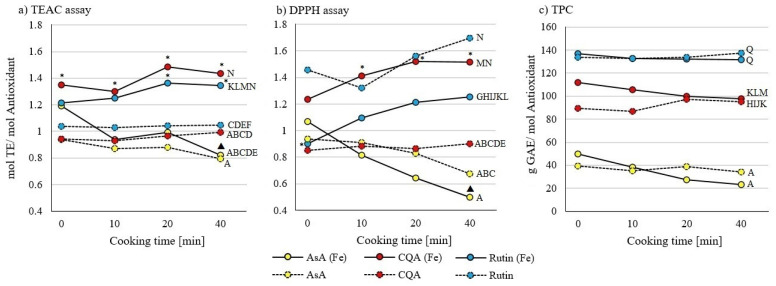
Influence of cooking time (0, 10, 20, and 40 min) on ascorbic acid (AsA; yellow), 5-caffeoylquinic acid (CQA; red), quercetin-3-rutinoside (Rutin; blue) with (solid lines) and without (dashed lines) iron (Fe) on antioxidant activity (AOA); standard deviation not shown. All samples were tested using (**a**) TEAC, (**b**) DPPH, and (**c**) TPC assays. Significant differences (*p* ≤ 0.05 by Tukey’s HSD test (*n* = 3)) with different cooking times of the same substance and between samples with and without iron are marked with an asterisk *. Differences to 0 min cooked samples of the same substance are marked with a triangle ▲. Letters indicate differences between the three substances as mean values over all measured times and are comparable to results of the same test assay in Figure 2 and Figure 3.

**Figure 2:**
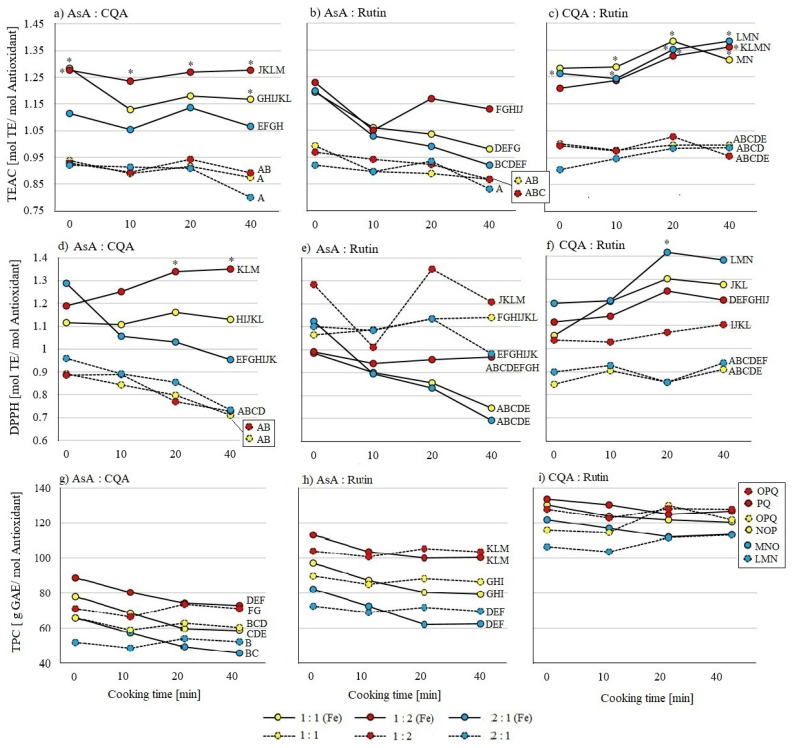
Influence of cooking time (0, 10, 20, and 40 min) on binary mixtures of ascorbic acid (AsA), 5-caffeoylquinic acid (CQA), quercetin-3-rutinoside (Rutin) with (solid lines) and without (dashed lines) iron (Fe) on antioxidant activity (AOA); standard deviation not shown. All samples tested using (**a**–**c**) TEAC, (**d**–**f**) DPPH, and (**g**–**i**) TPC assays. Colors indicate the different mixing ratios: equimolar mixtures are yellow, 1:2 ratios are red, and 2:1 ratios are blue. Significant differences (*p* ≤ 0.05 by Tukey’s HSD test (*n* = 3)) within different cooking times of the same substance and between samples with and without iron are marked with an asterisk *. Letters indicate differences between substance mixtures and ratios as mean values over all measured times and are comparable to results of the same test assay in Figure 1 and Figure 3.

**Figure 3:**
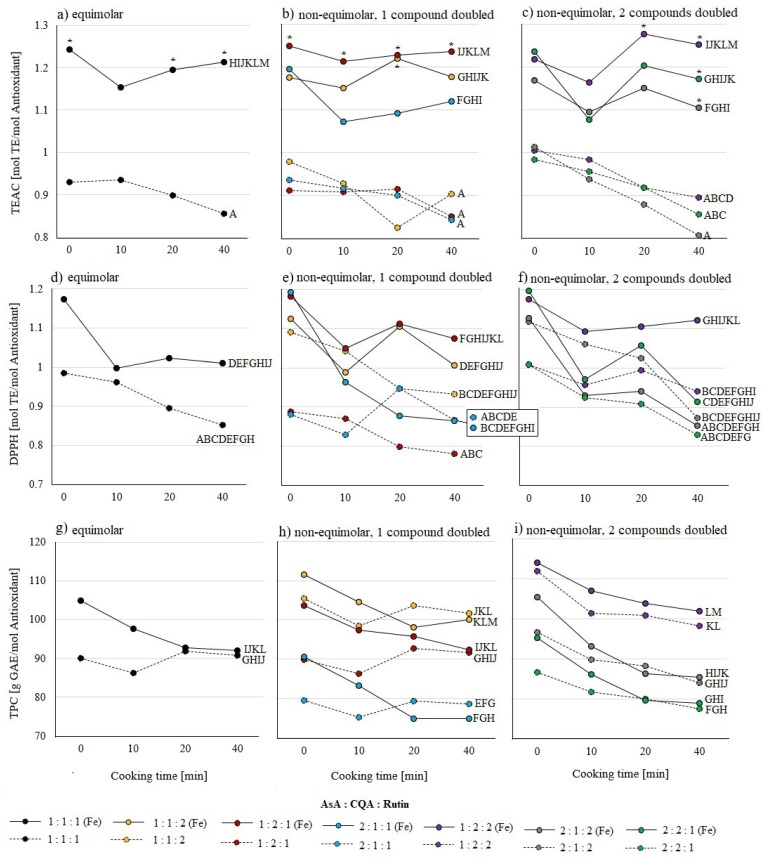
Influence of cooking time (0, 10, 20, and 40 min) on ternary mixtures of ascorbic acid (AsA), 5-caffeoylquinic acid (CQA), quercetin-3-rutinoside (Rutin) with (solid lines) and without (dashed lines) iron (Fe) on AOA; standard deviation not shown. All samples tested using (**a**–**c**) TEAC, (**d**–**f**) DPPH, and (**g**–**i**) TPC assays. Colors indicate different mixing ratios. Significant differences (*p* ≤ 0.05 by Tukey’s HSD test (*n* = 3)) with different exposure times of the same substance and between samples with and without iron are marked with an asterisk *. Letters indicate differences between substance mixtures and ratios as mean values over all measured times and are comparable to results of the same test assay in Figure 1 and Figure 2.

In none of the pure samples of ascorbic acid, 5-caffeoylquinic acid, or quercetin-3-rutinoside (Figure 1) or mixtures of them (Figure 2 and Figure 3) was an effect of cooking times between 0 and 40 min on AOA observed, except in the combination of ascorbic acid and iron. AOA was lower in TEAC and DPPH assays after 40 min of cooking, when compared to uncooked samples (Figure 1). Samples containing ascorbic acid tend to decrease in their AOA, while samples containing quercetin-3-rutinoside tend to increase their AOA with prolonged cooking time (Figure 1, Figure 2 and Figure 3).

### 2.2. Influence of Iron and Different Combinations of Ascorbic Acid, 5-Caffeoylquinic Acid, and Quercetin-3-Rutinoside Standards on Antioxidant Activity

Analyses with a TEAC assay (Figure 1a) showed that the AOA of pure ascorbic acid was lower than the AOA of quercetin-3-rutinoside. In the presence of iron, the AOA of ascorbic acid was even lower than the AOA of quercetin-3-rutinoside and 5-caffeoylquinic acid. Adding iron increased the AOA of quercetin-3-rutinoside and 5-caffeoylquinic acid. At a cooking time of 0 min, only the AOA of 5-affeoylquinic acid was higher in the presence and absence of iron. The DPPH assay (Figure 1b) detected higher AOA of quercetin-3-rutinoside in the absence of iron compared to 5-caffeoylquinic acid and ascorbic acid. The presence of iron led to an AOA increase for 5-caffeoylquinic acid, and an AOA decrease for quercetin-3-rutinoside samples. At 0 min cooking time only quercetin-3-rutinoside was higher in the absence of iron than in the presence of iron. Only in the TPC assay (Figure 1c) iron did not influence the AOA, and the order of the three substances stayed the same.

In binary mixtures, detected by the TEAC assay (Figure 2a–c), iron led to a significant or trending increase in AOA. Among samples without iron, no differences between AOA of mixtures or ratios were found. In the presence of iron at a cooking time of 0 min, the ratios 1:1 and 1:2 of ascorbic acid and 5-caffeylquinic acid, and a 2:1 ratio of the 5-caffeylquinic acid and queretin-3-rutinoside mixture, were higher in their AOA than their iron-free counterparts. In the DPPH assay (Figure 2d–f), the combination of ascorbic acid with 5-caffeoylquinic acid, as well as with quercetin-3-rutinoside, showed in the presence of iron in all ratios a higher AOA than the AOA of pure ascorbic acid. However, ascorbic acid combined with quercetin-3-rutinoside showed a higher AOA in the absence of iron, being comparable to pure quercetin-3-rutinoside. In the TPC assay (Figure 2g–i), the AOA of all three binary mixtures showed identical patterns. There was no influence of iron or cooking time on AOA. These results correspond with previous findings in pure substances (Figure 1): the lowest AOA was detected in ascorbic acid and 5-caffeoylquinic acid mixtures, followed by ascorbic acid with quercetin-3-rutinoside, and the highest AOA was found in the combination of 5-caffeoylquinic acid and quercetin-3-rutinoside.

In all ternary mixtures, no differences between ratios, regardless of the presence of iron, in TEAC and DPPH assays were found (Figure 3a–f). Higher AOAs were found in the TEAC assay (Figure 3a–c) in the presence of iron. At 0 min cooking time the AOAs of the equimolar mixture and the 1:2:1 ratio of ascorbic acid, 5-caffeoylquinic acid, and quercetin-3-rutinoside were higher in the presence of iron. The DPPH assay revealed higher AOA in samples with iron for the 1:2:1 ratio (Figure 3e). In accordance with binary mixtures, the TPC assay was neither influenced by iron nor by cooking time (Figure 3g–i). Furthermore, the equimolar mixture in the absence of iron had a lower AOA than the 1:2:2 ratio. In non-equimolar mixtures with one doubled compound, the 1:1:2 ratio was higher in AOA compared to the 2:1:1 ratio (Figure 3h) and in the non-equimolar with two doubled compounds, the 1:2:2 ratio was higher in AOA compared to the 2:1:2 and 2:2:1 ratio (Figure 3i) in the presence and absence of iron.

### 2.3. Synergistic and Antagonistic Effects of Antioxidant Activity

**Figure 4:**
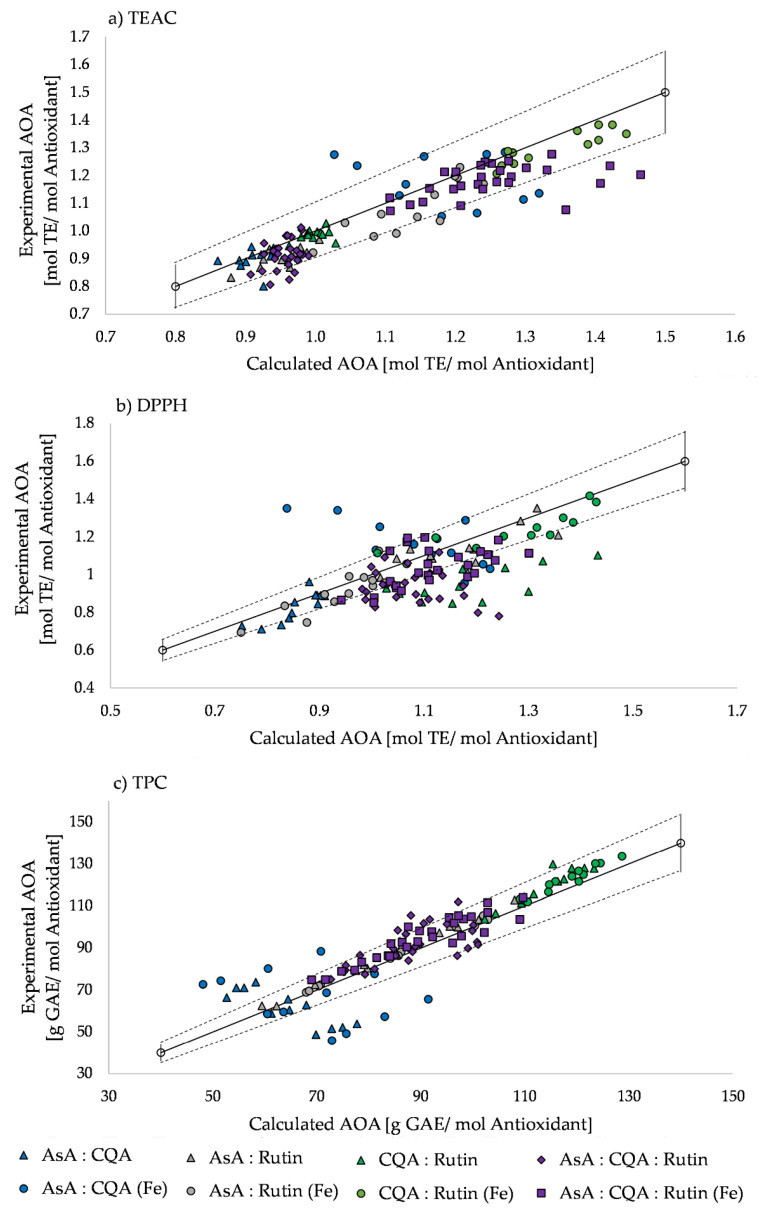
Synergistic and antagonistic effects based on calculated and experimental antioxidant activity (AOA) using (**a**) TEAC, (**b**) DPPH, and (**c**) TPC assays. Dots below the solid line indicate antagonistic, and above the solid line synergistic, effects. Colors indicate different mixtures, while samples with iron are marked with a dot, and those without iron are marked with a triangle. Dashed lines indicate weak antagonism (−10%)/synergism (+10%) with less than 10% interaction between the experimental and calculated AOA.

All test assays showed mainly weak synergistic and antagonistic effects with interactions below 10% (Figure 4). Noteworthily, among all used test assays, the 2:1 ratio of ascorbic acid and 5-caffeoylquinic acid in the presence of iron had strong synergistic effects, while the 1:2 ratio of ascorbic acid and 5-caffeoylquinic acid had strong antagonistic effects. In the TPC assay these phenomena also appeared in mixtures without iron. In the DPPH assay, another strong antagonistic effect was detected in all ratios of binary mixtures without iron containing 5-caffeoylquinic acid and quercetin-3-rutinoside (Figure 4b). For ternary ascorbic acid, 5-caffeoylquinic acid, and quercetin-3-rutinoside mixtures, the TEAC assay showed strong antagonistic effects for the 2:2:1 ratio with iron (Figure 4a). In ternary mixtures with iron the DPPH assay revealed strong antagonistic effects in cooked samples in ratios of 1:2:1, 1:1:2, 2:1:1, and 2:2:1, as well as in mixtures without iron in the ratios 1:1:2, 1:2:2, and 2:1:1 (Figure 4b). The TPC assay displayed strong synergistic effects in mixtures without iron in the 1:2:1 ratio (Figure 4c).

### 2.4. Total and Ionic iron

Ionic iron was added as an equimolar mixture of 50% ferric iron (Fe^3+^) and 50% ferrous iron (Fe^2+^) to the aforementioned pure, binary, and ternary mixtures. In all samples, the ratio of ferric iron shifted towards ferrous iron compared to the initially added equimolar ratio. Whenever ascorbic acid was present in samples, bound iron decreased with cooking time and subsequently vanished or stabilized (Table 1). A Pearson correlation on the TEAC assay data revealed a strong negative correlation (−0.641, *p* ≤ 2.2*10^−16^) between the AOA and ferrous iron ions, and a positive correlation between AOA and ferric iron (0.377, *p* ≤ 4.1*10^−9^) over all samples. Furthermore, the DPPH assay showed a negative correlation (−0.429, *p* ≤ 1.3*10^−11^) between AOA and ferrous iron ions. Meanwhile, AOA and ferric iron ions were only weakly correlated (0.225, *p* ≤ 0.0006). In the TPC assay, AOA and ferrous iron ions were strongly negatively correlated (−0.772, *p* ≤ 2.2*10^−16^), and AOA and ferric iron ions were strongly positively correlated (0.685, *p* ≤ 2.2*10^−16^).

In pure ascorbic acid samples, only traces of ferric iron were detectable, regardless of their cooking time. Additionally, the amount of ferrous iron increased, while bound iron decreased in all ascorbic acid samples after cooking. In 5-caffeoylquinic acid samples ferric iron decreased by 13.3% after 40 min of thermal processing, while at the same time ferrous iron slightly increased. Quercetin-3-rutinoside samples with iron resulted in an almost stable amount of ferrous iron. Bound iron increased with prolonged cooking time, while ferric iron decreased by 20.49% after 40 min of cooking (Table 1).

In the presence of ascorbic acid, 0 min cooked binary samples contained between 18.9% and 28.9% bound iron, which was disbound by cooking. Bound iron was found in binary mixtures after 20 and 40 min of cooking only when ascorbic acid was absent. Mixtures of ascorbic acid and 5-caffeoylquinic acid contained higher amount of ferrous iron than combinations of ascorbic acid and quercetin-3-rutinoside. The combination of 5-caffeoylquinic acid and quercetin-3-rutinoside in all ratios showed similar patterns, ferrous iron increased slightly, and ferric iron decreased with prolonged cooking time. Bound iron was found in this mixture only after 20 and 40 min of cooking (Table 2).

In ternary mixtures, the amount of ferrous iron was higher, and the amount of ferric iron lower, than initially spiked equimolar concentrations of each. The overall highest content of ferrous iron was found in samples when the concentration of ascorbic acid was doubled (ratios 2:1:1, 2:1:1, 2:2:1). The cooking process further increased the amount of ferrous iron and ferric iron, while bound iron decreased (Table 3).

### 2.5. Qualitative and Quantitative Analysis of the Substance Mixtures by HPLC

**Figure 5:**
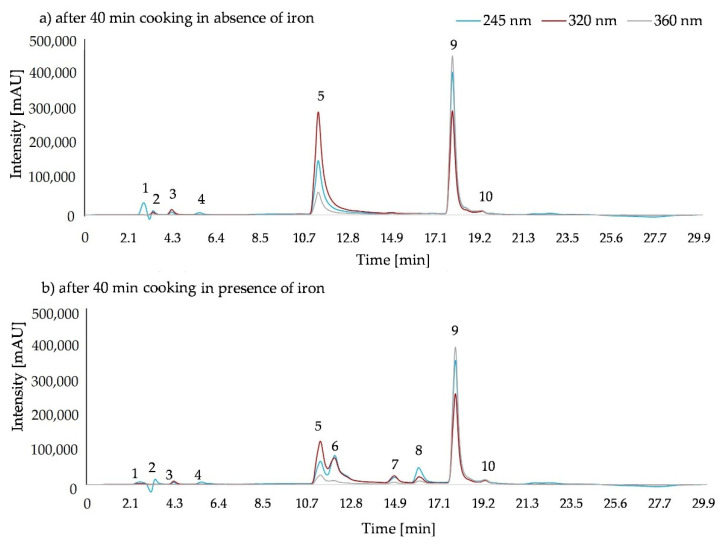
HPLC-DAD chromatograms of the 1:1:1 ratio of ascorbic acid, 5-caffeoylquinic acid, and quercetin-3-rutinoside (**a**) after 40 min cooking in the absence of iron, and (**b**) after 40 min cooking in the presence of iron. 1: ascorbic acid, 2: insert peak, 3: ascorbic acid derivate, 4: ascorbic acid derivate, 5: 5-caffeoylquinic acid, 6: caffeic acid; 7: 5-caffeoylquinic acid derivate, 8: quercetin-3-rutinoside derivate, 9: quercetin-3-rutinoside, 10: impurity of the quercetin-3-rutinoside standard.

HPLC data showed that in all 0 min cooked samples, in the presence and absence of iron, only the initially inserted substances of ascorbic acid, 5-caffeoylquinic acid, and quercetin-3-rutinoside were present (data not shown). After cooking for 40 min two additional peaks (peaks 3 and 4) derived from ascorbic acid, regardless of the presence of iron (Figure 5). In the presence of iron, two new products of 5-caffeoylquinic acid (peaks 6 and 7) and one from quercetin-3-rutinoside (peak 8) were detected (Figure 5b).

In the absence of iron, ascorbic acid concentrations decreased in all mixtures in a dose–response relation from an initial concentration of 0.3 mM after 40 min of cooking to 26.79% of the initial concentration and with an initial 0.2 mM between 44.08% and 51.67%, with an initial 0.15 mM between 60.49% and 65.47%, with an initial 0.1 mM 77.84% and 86.41% and increasing up to 90.05% with an initial 0.06 mM (Table S1).

In the presence of iron, only in pure ascorbic acid samples a decrease in concentration was found after 40 min of cooking, being higher compared to the ascorbic acid sample without iron (Table S1). Contrary to the ascorbic acid samples without iron, higher ascorbic acid concentrations led to higher degradation ratios (Table S1). In all binary mixtures, only ascorbic acid concentrations decreased in 0 min cooked samples. After 40 min of cooking, a decrease in 5-caffeoylquinic acid or quercetin-3-rutinoside concentration was found when combined with ascorbic acid. Both substances minimize the decrease in ascorbic acid concentration. Higher 5-caffeoylquinic acid concentrations led to minimizing the ascorbic acid degradation. In binary mixtures of 5-caffeoylquinic acid and quercetin-3-rutinoside, 5-caffeoylquinic acid decreased in its concentration, while quercetin-3-rutinoside stayed stable after 40 min of cooking. In ternary mixtures, quercetin-3-rutinoside stayed stable after 40 min of cooking, while ascorbic acid and 5-caffeoylquinic acid concentrations decreased.

## 3. Discussion

### 3.1. Structure–Activity Relationship of Ascorbic Acid, 5-Caffeoylquinic Acid, and Quercetin-3-Rutinoside

A first indicator of the AOA, measured for different substances dependent on the structure–activity relationship, resulted from the quantity of functional groups. Comparing all three analyzed substances, ascorbic acid had the lowest AOA, followed by 5-caffeoylquinic acid, while quercetin-3-rutinoside reached the highest AOA. Csepregi et al. [14] found the same order when comparing the AOA of these three compounds. This ranking may be explained by the total amount of hydroxy groups: quercetin-3-rutinoside has ten, 5-caffeoylquinic acid has five, and ascorbic acid has four. For flavonoids, the total amount of hydroxy groups and its impact on mechanisms of AOA was previously shown by Burda and Oleszek [15]. Hydroxy groups are particularly valuable in enediol structures, as they can easily oxidize to diketones [8]. In the analyzed substances the endiol structure is also important for the ability to form complexes with metal ions [16,17,18]. The endiol structure occurs in quercetin-3-rutinoside and 5-caffeoylquinic acid molecules in the phenol ring, which may also influence the stronger AOA of these compounds. Further studies found that the AOA may also be influenced by other molecule structures. Phenolic acids are possibly affected by the carboxylic acid groups, e.g., hydroxyphenylacetic acid (R-CH=CH-COOH) is a weaker electron-withdrawing group, compared to hydroxycinnamic acid (R-CH_2_-COOH) such as caffeic acid in 5-caffeoylquinic acid [19]. In flavonoids, aglycones had higher AOA than the corresponding glycosides [20,21], in the case of quercetin-3-rutinoside, a glycoside, the AOA of the aglycone quercetin is consequently higher [14]. There are many different functional groups that can influence the AOA and, consequently, it is important to use different test assays with different mechanisms, such as single-electron transfer (SET), hydrogen atom transfer (HAT), and sequential proton-loss electron transfer (SPLET) for detection. Each mechanism and even the used assay reagent can detect different structures of bioactive compounds [22].

### 3.2. Influence of Thermal Processing and Interaction of Structurally Different Antioxidants on the Antioxidant Activity in the Absence of the Mineral Iron

Stable AOA over an extended time of 40 min of thermal processing indicates that thermal degradation is of less importance than initially hypothesized. Furthermore, HPLC data showed that pure 5-caffeoylquinic acid and quercetin-3-rutinoside were stable, with a maximum degradation of 20%, during thermal processing. Previous studies proved the stability of 5-caffeoylquinic acid up to the normal boiling point of water [23], while Dawidowicz and Typek [24] found nine derivative compounds, after heating 5-caffeoylquinic acid for 5 h under reflux. For ascorbic acid concentration, a degradation after 40 min of cooking was found. Influencing factors, resulting in ascorbic acid degradation without iron in an aqueous solution, could be pH values, light exposure, oxidation, temperature, and different concentrations [25,26]. In this study the factors temperature and concentration most likely played the main role, as oxidative processes were reduced to a minimum due to minimal gas space in the microtubes. It is possible that the remaining gas phase is still enough for degradation under aerobic conditions. The different conditions result in different products [27,28], so in this study, after 40 min of cooking, products of both conditions could be found. Yuan and Chen [28] reported that furfural, 2-furoic acid, 3-hydroxy-2-pyrrone, and an unknown substance are major degradation products of ascorbic acid in an aqueous solution depending on pH value. Shinoda et al. [29,30] found in orange juice the degradation products furfural, 2-furoic acid, 5-hydroxymaltol, 3-hydroxy-2-pyrrone, and 5-(hydroxymethyl)furfural. Hsu et al. [31] analyzed ascorbic acid in ethanolic solutions and detected 2-furoic acid and 3-hydroxy-2-pyrrone. Depending on aerobic or anerobic conditions, the detected ascorbic acid derivatives (peaks 3 and 4) might be furfural, 2-furoic acid, or 3-hydroxy-2-pyrrone or intermediates. The ascorbic acid decreased in a dose–response relation, and higher concentrations of ascorbic acid showed lower degradation ratios during cooking. Ascorbic acid seems to stabilize itself in higher concentrations, presumably due to hydrogen bonds and van de Waals energy [32].

In binary and ternary mixtures, mainly additional effects on AOA were found. In binary mixtures, if the substance with higher AOA increased in its concentration, the AOA of their combination also increased. Other studies also found additive effects between (+)-catechin (200 µm) with ascorbic acid (50–200 mg/L) [33], and between binary mixtures of different monoterpenes [34]. Only in the DPPH assay antagonistic effects were found in 5-caffeoylquinic acid combined with quercetin-3-rutinoside and in ternary mixtures with doubled quercetin-3-rutinoside concentrations. This could be caused by the orientation of the molecules in space, especially quercetin-3-rutinoside, as a quite large molecule, and the steric accessibility of the DPPH radical molecule [35]. In the TPC assay, the combination of ascorbic acid and 5-caffeoylquinic acid resulted in strong antagonistic effects in a 1:2 ratio, while reversed mixtures with a 2:1 ratio resulted in strong synergistic effects, exceeding the sum of the respective AOAs. Double concentration ascorbic acid may give, under these conditions, an additional boost to AOA, due to self-stabilization followed by the stabilization of other molecules, demonstrated by 5-caffeylquinic acid in this experiment. In pomegranate–nectarine juice, between the natural phenols and ascorbic acid, the same interaction was found, while in grape juice increasing antagonistic effects by increasing ascorbic acid concentration were observed [36]. These results suggest that mixtures of ascorbic acid, 5-caffeoylquinic, and quercetin-3-rutinoside achieve the highest AOA potential when most potent antioxidants are abundantly available.

### 3.3. Influence of the Mineral Iron

The addition of iron to the samples had the most influence on changes in their AOA. Contrary to the hypothesis, based on the pro-oxidative activity of iron, the AOA increased, compared to the same substance or substance mixture without iron. Iron may act like a catalyst itself or forms metal chelates, which are effective catalysts. The changed stoichiometry of the chelates can form additional radical-scavenging metal centers [18], which explains the increased AOA. Further tests showed that reduced ferrous iron (50–100%, *v/v*) itself interacts with the TEAC (0.266–0.538 mol TE/mol iron), DPPH (0.210–0.495 mol TE/mol iron), and TPC (16.65–31.82 g GAE/mol iron) assay reagents, while oxidized ferric iron does not (data not shown). In the presence of iron, 5-caffeoylquinic acid had the highest AOA, followed by quercetin-3-rutinoside and ascorbic acid in TEAC and DPPH assays. This may be due to the changed stoichiometry by metal chelation. The AOA ranking detected by the TPC assay stayed the same, as in the absence of iron: quercetin-3-rutinoside > 5-caffeoylquinic acid > ascorbic acid. However, the addition of iron to the mixtures had an influence on TEAC and DPPH assay results, while TPC assays were widely unaffected. For this reason, it is important to use different test assays with different working mechanisms when working with iron-rich samples.

Only in the DPPH assay the AOA of pure quercetin-3-rutinoside, and in combination with ascorbic acid, equimolar or non-equimolar with doubled quercetin-3-rutinoside concentrations, did have lower values in the presence of iron. Boligon et al. [35] explained that the DPPH assay detects smaller antioxidants better, due to the steric accessibility of these radicals. Presumably, quercetin-3-rutinoside with the two binding sides can build quite large complexes with iron, and thus be inaccessible for the assay. As mentioned by Kejíc et al. [8], this might be explained by the formation of supramolecular complexes via a coordination of metal ions. In combination with ascorbic acid, which is able to build mixed-valence complexes in a 1:2 ratio [12], presumably, these larger mixed complexes cannot interact with the DPPH radical. Contrary to the results in this study, other studies [18,37,38] found, by using the DPPH assay, that quercetin-3-rutinoside complexes are more effective antioxidants than pure quercetin-3-rutinoside. It is known that chelate complexes of flavonoids and metal ions can negate the radical activity of complexed metal ions [39]. The only difference was that in this study ammonium iron (II) sulfate was used, instead of iron (II) chloride or sulfate, used in the work of Symonowics and Kolandek [39].

Synergistic effects between ascorbic acid and 5-caffeoylquinic acid in the presence of iron in the 2:1 ratio, and antagonistic effects in the 1:2 ratio, were found. The combination of 5-caffeoylquinic acid with iron was not recommended in vivo [6]. However, this study showed that, in vitro, the doubled amount of ascorbic acid in comparison to 5-caffeoylquinic acid can even have synergistic effects on AOA. Further investigations on ratios could show whether positive effects can also be achieved in vivo.

According to the HPLC data, adding iron to the sample will have a minimal effect on its catalytic activity, as only the ascorbic acid concentration in 0 min cooked samples decreased. In the cooked ascorbic acid samples, contrary to samples without iron, higher ascorbic acid concentrations led to a higher degradation ratio. 5-Caffeoylquinic acid and quercetin-3-rutinoside need the additional factors temperature and time, as well as interactions in mixtures, for the catalytical activity of iron to work. Quercetin-3-rutinoside, in the presence of iron, only decreased in binary mixtures with ascorbic acid after thermal processing. This could be due to the fact that the flavonoid acts as a primary antioxidant and then the resulting compound radical reacts with ascorbic acid, regenerating the original compound [18]. 5-Caffeoylquinic acid seem to be special, because it protects quercetin-3-rutinoside, while ascorbic acid is not able to protect quercetin-3-rutinoside. Hence, 5-caffeoylquinic acid may be the key molecule for stabilizing the system in combination with iron and thermal processing. This hypothesis of 5-caffeoylquinic acid as a stabilizing molecule was further confirmed in the ternary mixtures. Here, quercetin-3-rutinoside was always stabilized by 5-caffeoylquinic acid, so that even in the presence of ascorbic acid no reduction in quercetin-3-rutinoside concentration was observed. In another study, 5-caffeoylquinic acid demonstrated protective properties against degradation of anthocyanins trough a co-pigmentation mechanism [40]. New degradation products, with possibly higher AOA, appeared from all three substances in the presence of iron. Caffeic acid (peak 6) was identified as a 5-caffeoylquinic acid breakdown product (data not shown). This led to the assumption that the other substance may be quinic acid or one of the nine possible derivatives described by Dawidowicz and Typek [24]: quinic acid; (1S,3R,4R,5R)-5-[3-(3,4-dihydroxyphenyl)-2-hydroxypropanoyl]-1,4,5-trihydroxy-cyclohexanecarboxylic acid; (1S,3R,4R,5R)-5-[3-(3,4-dihydroxyphenyl)-3-hydroxypropanoyl]-1,4,5 trihydroxycyclohexanecarboxylic acid; trans 3-*O*-caffeoylquinic acid; trans 5-*O*-caffeoylquinic acid; trans 4-*O*-caffeoylquinic acid; caffeic acid; cis-5-*O*-caffeoylquinic acid; 4,5-dicaffeoylquinic acid. For quercetin-3-rutinoside, a degradation product appears in the chromatogram, which could not be identified.

### 3.4. Ability to Form Chelates with Ferric (Fe^3+^) and Ferrous Iron (Fe^2+^)

In all samples containing ascorbic acid, ferric iron was reduced to ferrous iron. In this process, ascorbic acid takes an electron from the ferric iron and reduces it to ferrous iron and becomes a radical itself. The unstable radical converts rapidly to dehydroascorbic acid and further degradation products [12]. Due to missing hydroxy peroxide, a reaction back to ferric iron via the Fenton cycle is not possible. In 0 min cooked binary mixtures, more than 20 % of iron was bound in the presence of ascorbic acid. It is known that ascorbic acid forms complexes with iron species and other metal ions by chelation via the 3-*O* and 2-*O* nuclei following hydrogen displacement from the 3-OH and 2-OH groups [16,17,41]. It can also form mixed valence iron–ascorbate complexes [42]. However, ascorbic acid is a weak chelating agent and, after cooking, only traces of bound iron were detected in pure and mixed samples when ascorbic acid was present. Furthermore, due to the cooking process, ascorbic acid breaks down to degradation products, which seem unable to chelate with iron.

5-Caffeoylquinic acid is a relatively poor reductant. Ferric iron was reduced by 5-caffeoylquinic acid, and with prolonged cooking, reduction increased. 5-Caffeoylquinic acid chelates with ferric iron in a ligand to metal charge transfer [17]. 5-Caffeoylquinic acid carries one possible binding side at the 3,4 endiol structure of the caffeic acid. This could be an indicator as to why caffeic acid is the bioactive part of 5-caffeoylquinic acid, while quinic acid has almost no AOA [43]. Endiols inhibited OH formation due to the formation of an iron complex [41] in a one-to-one ratio [44,45]. Lamy et al. [46] conclude that 5-caffeoylquinic acid forms monomeric complexes, whereas Kiss et al. [47] found oligomeric species. Contrary to previous studies [17,48], only traces of bound iron were found in the presence of 5-caffeoylquinic acid pre- and post-thermal processing. This can be explained by the neutral pH conditions in this study, while other studies worked in an acidic medium, based on a pH value from 1–2.5 in the human stomach [48]. A black precipitate was found in stored samples after several hours, which is an indicator of 5-caffeoylicquinic acid–ferric iron complexes. Recently mixed samples were used for the analysis, so the formation of these complexes at a neutral pH requires a longer period of time. Iron complexes with caffeic acid showed little scavenging activity [49]. Furthermore, there is no spectrophotometric evidence for a reaction between quinic acid and ferric iron [48]. Contrary to 5-caffeicquinic acid, in the quercetin-3-rutinoside sample, bound iron was found, even after thermal processing.

Quercetin-3-rutinoside reduced ferric iron to ferrous iron with a minimum increase by a prolonged cooking time. This moderate reducing activity of quercetin-3-rutinoside was previously described by Mira et al. [50]. The ferric reducing activity of quercetin-3-rutinoside was detected in 3-rutinoside, 5,7,3′,4′-OH [50]. The moderate interaction with ferric iron can be explained by a lower number of -OH groups, which resulted in lower negative charge density at the chelation side [50]. Flavonoids can chelate with metal ions at three potential coordination sides: (i) between 5-hydroxy and 4-carbonyl groups, (ii) between 3-hydroxy and 4-carbonyl groups, and (iii) between 3′,4′-hydroxy groups in B ring [39]. Quercetin-3-rutinoside uses the binding sides (i) and (iii) [9], and at the 3-hydroxy group the rutinoside is attached. Spectral data even showed that metal ions only bound to the 3′,4′-hydroxy group [18]. Chelates are more effective with iron in its bivalent form [50]. In binary mixtures, quercetin-3-rutinoside is not able to form complexes when ascorbic acid is present, contrary in ternary mixtures. If 5-caffeoylquinic acid is present, small amounts of bound iron are found. This indicates that 5-caffeoylquinic protects quercetin-3-rutinoside molecules in the presence of ascorbic acid, so quercetin-3-rutinoside can form complexes with iron.

## 4. Materials and Methods

### 4.1. Chemicals

ABTS^•+^ (2,2′-azinobis (3-ethylbenzothiazoline-6-sulfonic acid) diammonium salt) (≥98%) was obtained from Sigma-Aldrich (Steinheim, Germany), DPPH^•^ (2,2-diphenyll-1-picryhydrazyl) radical (95%) and Trolox^®^ (97%) were obtained from Thermo Fisher (Kandel, Germany). Folin–Ciocalteu phenol reagent was purchased from Merck (Darmstadt, Germany). HPLC grade methanol, acetonitrile (HPLC grade), glacial acetic acid (100%, p.a.), sodium acetate trihydrate (≥99.5% p.a.), potassium thiocyanate (≥98.5%, p.a., ACS), 2,2′-dipyridyl (≥95%), hydrochloric acid (≥25%, p.a., ISO), gallic acid monohydrate (≥99%), potassium persulfate (≥99%), rutin trihydrate (working standard), chlorogenic acid (working standard), L-(+)-ascorbic acid (working standard), sodium carbonate (≥99%), ferric ammonium sulfate dodecahydrate, and ferrous ammonium sulfate hexahydrate were purchased from Carl Roth (Karlsruhe, Germany).

### 4.2. Samples

Aqueous solutions and mixtures of authentic standards of ascorbic acid, 5-caffeoylquinic acid, and quercetin-3-rutinoside were prepared as pure solutions and mixtures in different ratios. All possible binary mixtures were made in equimolar ratios, and non-equimolar ratios with one compound doubled. Ternary mixtures were also prepared in equimolar ratios, as well as in non-equimolar ratios with one or two compounds doubled, respectively. The antioxidants’ final concentrations were 0.3 mM for each test solution. Additionally, all experiments were repeated after the addition 0.3 mM iron (0.15 mM ferrous iron and 0.15 mM ferric iron) in total per mixture. The chosen amounts of antioxidants and iron are not based on physiological or food levels, they are based on molar masses to study the effect of molecular interaction. Consequently, an equimolar ratio between total antioxidants and iron was established. All mixtures were cooked for 0, 10, 20, and 40 min in boiling water, and afterwards cooled on ice to stop the heating process. This was done for three independent replicates.

### 4.3. Photometric Measurements

Antioxidants (pure or mixed) in the absence and presence of iron were measured for their total reducing activity and antioxidant activity in three independent technical replicates using a high-throughput method in 96-well plates (Synergy™ HTX Multi-Mode Microplate Reader, BioTek Instruments, Winooski, VT, USA). Different test assays were used, because of the different reaction mechanisms: single-electron transfer (SET) and hydrogen atom transfer (HAT). While the TEAC and TPC assay are based on SET [17,18], for the DPPH test assay, the literature is not quite clear if it based on SET, HAT, or even a combination of these two mechanisms [21,22,23]. A recent study of Foti [51] discovered that phenols can react with DPPH via sequential proton-loss electron transfer (SPLET), a combination of the two mechanisms. Factors, such as medium polarity and ionization potential, influence the predominant mechanism.

#### 4.3.1. Total Phenolic Content (TPC)

Total phenol content (TPC) was determined using the Folin–Ciocalteu method in a 96-well plate, being previously described by Bobo-García et al. [52] with some modifications. Briefly, 10 µL Folin–Ciocalteu reagents were mixed with 50 µL sample and afterwards 100 µL Na_2_CO_3_ was added. The 96-well plate was incubated at 37 °C (±0.2 °C) and with constant orbital shaking at a moderate speed (237 cpm, 4 mm) for 14 min. After a 1 min resting period, the absorbance was measured at 736 nm. Results were expressed as gallic acid equivalents (mg GAE/ mol Antioxidant), using a standard curve ranging from 5.97 to 59.7 µg gallic acid/mL (R^2^ > 0.99). The common name of this test is misleading, because the Folin–Ciocalteu reagent also reacts on non-phenolics, such as vitamins and minerals [22]. It describes better the “total reducing activity” of bioactive compounds.

#### 4.3.2. Trolox Equivalent Antioxidant Capacity (TEAC)

The antioxidant activity was determined using the TEAC assay in a 96-well plate with some modifications. A stock solution with 9.6 mg ABTS and 1.66 mg potassium persulfate filled up with H_2_O to 25 mL was prepared and incubated in the dark at room temperature for 12–16 h. From this stock solution, a TEAC working solution, containing 5 mL stock solution filled up to 25 mL with 100% MeOH, was prepared.

Briefly, 10 µL of the sample was mixed with 150 µL of the TEAC working solution. After a 5 min incubation, the plate was shaken orbitally at a moderate speed for 1 min, followed by a 1 min resting period. The absorbance was measured at a wavelength of 734 nm. The TEAC was expressed as Trolox equivalents (mol TE/mol Antioxidant), using a standard curve ranging from 0.025–0.8 mM Trolox (R^2^ > 0.98).

#### 4.3.3. DPPH^•^ Radical Scavenging

The antioxidant activity was determined using the modified DPPH method for 96- well plates. A DPPH working solution with 7.88 mg DPPH filled up to 100 mL was prepared. Briefly, 20 µL of the sample was mixed with 180 µL of the DPPH working solution and incubated in the dark for 28 min at room temperature. After 1 min orbital shaking at a moderate speed and a 1 min resting time, the absorbance was measured at a wavelength of 515 nm. Results were expressed as Trolox equivalents (mol TE/mol Antioxidant), using a standard curve ranging from 0.025–0.8 mM Trolox (R^2^ > 0.98).

### 4.4. Synergism and Antagonism

For analysis of synergistic and antagonistic effects of the antioxidant activity, a comparison of the results obtained experimentally with the theoretical values calculated by the sum of the effects of individual components at the corresponding concentration was made [53]. Synergism describes an interaction of two or more substances, so that the combined action is greater than the sum of each acting separately. Contrary to this, antagonism is a phenomenon where the interaction of two or more substances in combination have an overall effect that is less than the sum of their individual effects.

### 4.5. Determination of Ionic Iron

The colorimetric determination of ferrous and ferric iron was modified according to Niedzielski et al. [54] for 96-well plates. Briefly, for ferrous iron detection, 20 µL acetate buffer (90 g sodium acetate trihydrate and 48 g acetic acid glacial filled up to 200 mL) and 20 µL 2,2′dipyridyl (0.5%, *m*/*m*) and, for ferric iron detection, 20 µL hydrochloric acid (2 M) and 20 µL potassium thiocyanate (5%, *m/m*) were pipetted into a 200 µL sample in the 96-well plate, incubated there for 10 min at room temperature, and the absorbance was measured for ferrous iron at 520 nm and for ferric iron at 470 nm. Results were expressed in mM ionic iron/mM total iron, using a standard curve ranging for ferrous iron from 0.024–0.214 mM (R^2^ > 0.99) and for ferric iron from 0.005–0.178 mM (R^2^ > 0.99). The difference between total iron and ionic iron, the sum of ferrous and ferric iron, is the bound iron.

### 4.6. HPLC-DAD

To quantify the antioxidants ascorbic acid, 5-caffeoylquinic acid, and quercetin-3-rutinoside and degradation products thereof in the same extracts used for the photometric measurements, a Shimadzu Prominence 20 high-performance liquid chromatography (HPLC) system equipped with a refrigerated SIL-20AC HT autosampler, CTO-10AS VP column oven, DGU-20A5 degasser, LC-20 AT liquid chromatograph quaternary pump, and an SPD-M20A diode array detector (DAD) was used. As a column for separation, a Supelco^®^ Ascentis^®^ Express F5 column (150 × 3.0 mm, 5 µm) equipped with a Supelco^®^ Guard column (5 × 3.0 mm, 5 µm) and a 0.2 micron SST Frit for UltraLine was used. The column temperature was set to 30 °C. UV detection was at 245 nm for ascorbic acid, 320 nm for 5-caffeoylquinic, and 360 nm for quercetin-3-rutinoside. The mobile phase consisted of Eluent A (1% acetic acid (*v/v*), pH 2.5) and Eluent B (100% ACN). The separation was achieved using the following gradient program: 0–2.5 min, 5% B; 2.5–15 min, 5–20% B; 15–20 min, 20% B; 20–22.5 min, 20–5% B; 22.5–30 min, 5% B. The flow rate was 0.3 mL/min, and the sample injection volume was 30 µL. Standard calibration curves for the three substances 5-caffeoylquinic (0.5–0.15 µM; R^2^ > 0.99), ascorbic acid (0.35–0.025 µM; R^2^ > 0.99), and quercetin-3-rutinoside (0.35–0.025 µM; R^2^ > 0.99) were prepared. Derived compounds were tentatively identified by analyzing the pure standards in the presence and absence of iron pre- and post-thermal processing. Therefore, the new peak must derive from the insert standard. Furthermore, selected mixtures were measured via HPLC-MS to verify the proposed structure.

### 4.7. Statistical Analysis

Microsoft Excel 2016 (Microsoft, Redmond, USA) and R Statistics (version 3.6.3, Holding the Windsock, 2020) were used for biostatistics tests and presenting and drawing data results. Inferential statistics for assessing and linking treatments were carried out by using three-way analysis of variance (ANOVA), a post hoc Tukey’s HSD test, and Pearson correlation. The R packages used were ggplot2 [55], emmeans [56], and multcomp [57].

## 5. Conclusions

Based on the above-described findings, the AOA of ascorbic acid, 5-caffeoylquinic acid, and quercetin-3-rutinoside was influenced by their molecule structure, concentration, ratio, and interactions with other antioxidants and iron. Interaction especially seems to play a role in AOA when combining ascorbic acid and 5-caffeoylquinic acid. Here, synergistic and antagonistic effects were detected. Temperature had a minimal influence on AOA, while at the same time temperature influenced the stability of all antioxidants in certain mixtures, especially in the presence of iron. Only the ascorbic acid concentration decreased in the absence of iron with prolonged cooking time and 5-caffeoylquinic acid concentration decreased only in the presence of iron, while quercetin-3-rutinoside concentration decreased only in combination with ascorbic acid in the presence of iron. In combination with iron, 5-caffeoylquinic acid was able to protect other molecules from being reduced in their concentration by thermic processing.

In plants, combinations of ascorbic acid, 5-caffeoylquinic acid, and quercetin-3-rutinoside are not only possible but common. Hence, these results give basic knowledge on the processes that occur during the cooking of vegetables. Food matrices are more complex and contain countless bioactive compounds, including enzymes, other minerals, or acids, that change reaction conditions or are reactants themselves. Those complex interactions are far beyond the scope of this study and beneficial concentrations and interactions of antioxidants in cooked vegetables have to be addressed in future.

## Figures and Tables

**Table 1:** Measured ionic iron species, ferric iron (Fe^3+^) and ferrous iron (Fe^2+^), and calculated bound iron after mixing with ascorbic acid, 5-caffeoylquinic acid, and quercetin-3-rutinoside, and cooking for 0, 10, 20, and 40 min with equimolar concentrations of ferrous and ferric iron in percent; traces ≤ 5%.

Substance	Time [min]	Ferrous Iron [%]	Ferric Iron [%] ^a^	Bound Iron [%] *
Ascorbic acid (AsA)	0	77.9 ± 0.8	traces	21.17 ± 0.4
	10	98.9 ± 0.8	traces	traces
	20	97.1 ± 0.9	traces	traces
	40	96.7 ± 1.1	traces	traces
5-Caffeoylquinic acid (CQA)	0	57.73 ± 3.8	42.27 ± 3.8	-
	10	64.04 ± 4.2	35.96 ± 4.2	-
	20	70.51 ± 3.3	26.78 ± 2.1	traces
	40	67.80 ± 2.4	28.97 ± 4.9	traces
Quercetin-3-rutinoside (Rutin)	0	55.02 ± 2.1	44.98 ± 2.1	-
	10	55.25 ± 3.2	42.20 ± 3.7	traces
	20	56.65 ±4.3	27.38 ± 5.2	15.97 ± 2.1
	40	54.77 ±4.2	24.49 ± 4.3	20.74 ± 8.3

^a^ LOD_ferric iron_ = 0.45%; LOQ_ferric iron_ = 1.36%. * Bound iron = 100 − ferrous iron + ferric iron.

**Table 2:** Measured ionic iron species, ferric iron (Fe^3+^) and ferrous iron (Fe^2+^), and calculated bound iron after mixing with the double standard mixtures of ascorbic acid (AsA), 5-caffeoylquinic (CQA), and quercetin-3-rutinoside (Rutin), and cooking for 0, 10, 20, and 40 min with equimolar concentrations of ferrous and ferric iron in percent; traces ≤ 5%.

Substance	Time [min]	Ferrous Iron [%]	Ferric Iron [%] ^a^	Bound Iron [%] *
1 AsA: 1 CQA	0	68.6 ± 0.8	traces	28.9 ± 0.2
	10	97.4 ± 0.7	traces	traces
	20	94.1 ± 0.4	5.5 ± 1.0	traces
	40	92.6 ± 2.6	7.4 ± 2.6	-
1 AsA: 2 CQA	0	73.2 ± 8.7	5.4 ± 1.0	21.4 ± 7.8
	10	91.2 ± 0.5	8.8 ± 0.5	-
	20	92.0 ± 0.8	8.0 ± 0.8	-
	40	91.0 ± 2.1	8.6 ± 2.4	traces
2 AsA: 1 CQA	0	79.5 ± 11.5	traces	18.9 ± 11.2
	10	98.8 ± 0.8	traces	traces
	20	96.5 ± 0.7	traces	traces
	40	93.9 ± 0.8	5.8 ± 0.9	traces
1 AsA: 1 Rutin	0	74.5 ± 7.7	traces	23.1 ± 7.1
	10	90.9 ± 3.0	9.1 ± 3.0	-
	20	86.4 ± 0.2	13.6 ± 0.2	-
	40	81.1 ± 5.7	18.8 ± 5.7	traces
1 AsA: 2 Rutin	0	73.0 ± 7.0	5.9 ± 1.4	21.1 ± 6.0
	10	80.8 ± 1.3	19.2 ± 1.3	-
	20	79.3 ± 0.6	20.7 ± 0.6	-
	40	76.7 ± 4.5	21.8 ± 6.2	traces
2 AsA: 1 Rutin	0	76.2 ± 9.1	traces	22.0 ± 8.7
	10	96.5 ± 2.3	traces	traces
	20	89.7 ± 0.1	10.3 ± 0.1	-
	40	84.9 ± 3.5	15.1 ± 3.5	-
1 CQA: 1 Rutin	0	56.6 ± 1.8	43.4 ± 1.8	-
	10	60.4 ± 3.8	38.3 ± 3.8	traces
	20	65.0 ± 4.0	29.4 ± 2.2	5.6 ± 3.2
	40	61.6 ± 1.2	33.1 ± 5.4	5.3 ± 4.3
1 CQA: 2 Rutin	0	55.2 ± 2.4	44.8 ± 2.4	-
	10	59.4 ± 4.0	39.7 ± 3.8	traces
	20	63.3 ± 3.9	28.9 ± 3.3	7.8 ± 5.0
	40	60.7 ± 3.0	30.5 ± 3.7	8.8 ± 6.6
2 CQA: 1 Rutin	0	56.1 ± 3.2	43.9 ± 3.2	-
	10	61.4 ± 3.6	38.4 ± 3.5	traces
	20	66.5 ± 3.9	26.9 ± 1.3	6.6 ± 2.6
	40	62.8 ± 3.2	29.5 ± 8.0	7.7 ± 5.6

^a^ LOD_ferric iron_ = 0.45%; LOQ_ferric iron_ = 1.36%. * Bound iron = 100 − ferrous iron + ferric iron.

**Table 3:** Measured ionic iron species, ferric iron (Fe^3+^) and ferrous iron (Fe^2+^), and calculated bound iron after mixing with the ternary standard mixtures of ascorbic acid (AsA), 5-caffeoylquinic (CQA), and quercetin-3-rutinoside (Rutin), and cooking for 0, 10, 20, and 40 min with equimolar concentrations of ferrous and ferric iron in percent; traces ≤ 5 %.

Substance	Time [min]	Ferrous Iron [%]	Ferric Iron [%] ^a^	Bound Iron [%] *
1 AsA: 1 CQA: 1 Rutin	0	69.65 ± 7.8	6.94 ± 1.3	23.41 ± 7.0
	10	84.66 ± 1.2	15.34 ± 1.2	-
	20	84.10 ± 1.2	15.90 ± 1.2	-
	40	81.39 ± 3.3	16.30 ± 4.9	traces
1 AsA: 2 CQA: 1 Rutin	0	69.16 ± 6.2	12.04 ± 0.4	18.80 ± 6.3
	10	79.05 ± 0.8	20.95 ± 0.8	-
	20	78.93 ± 2.8	20.53 ± 2.5	traces
	40	74.91 ± 4.9	22.90 ± 6.5	traces
1 AsA: 1 CQA: 2 Rutin	0	69.62 ± 6.5	12.47 ± 0.5	17.91 ± 6.2
	10	77.88 ± 0.8	22.12 ± 0.8	-
	20	78.05 ± 0.6	21.25 ± 1.4	traces
	40	71.91 ± 5.6	25.41 ± 7.5	traces
2 AsA: 1 CQA: 1 Rutin	0	73.37 ± 8.9	traces	23.87 ± 8.3
	10	90.88 ± 2.1	9.12 ± 2.1	-
	20	88.78 ± 0.2	11.22 ± 0.2	-
	40	87.75 ± 0.7	12.25 ± 0.7	-
1 AsA: 2 CQA: 2 Rutin	0	67.85 ± 6.3	18.60 ± 0.8	13.55 ± 5.5
	10	74.33 ± 0.8	25.67 ± 0.8	-
	20	74.51 ± 1.0	23.40 ± 0.5	traces
	40	69.98 ± 4.3	24.64 ± 8.1	5.38 ± 3.8
2 AsA: 1 CQA: 2 Rutin	0	71.34 ± 8.3	5.34 ± 1.3	23.32 ± 7.5
	10	87.63 ± 2.0	12.37 ± 2.0	-
	20	86.47 ± 0.5	13.25 ± 0.6	traces
	40	82.40 ± 4.1	17.36 ± 4.3	traces
2 AsA: 2 CQA: 1 Rutin	0	71.62 ± 0.8	5.11 ± 1.0	23.28 ± 7.3
	10	88.93 ± 0.3	11.07 ± 0.3	-
	20	89.31 ± 1.1	9.85 ± 2.0	traces
	40	85.34 ± 3.6	14.50 ± 3.8	traces

^a^ LOD_ferric iron_ = 0.45%; LOQ_ferric iron_ = 1.36%. * Bound iron = 100 − ferrous iron + ferric iron.

## Data Availability

The data presented in this study are available on request from the corresponding author.

## Supplementary Materials

The following are available online, Table S1: Degradation ratio in percent measured by HPLC in presence and absence of iron. Values within ± 20% are marked as stable.

## Sample Availability

Samples are made from standards, purchasable at most laboratory resellers.
