# Supplementary material for: Interactions of Ascorbic Acid, 5-Caffeoylquinic Acid, and Quercetin-3-Rutinoside in the Presence and Absence of Iron during Thermal Processing and the Influence on Antioxidant Activity
Source: Molecules. 2021 Dec 20;26(24):7698. doi: 10.3390/molecules26247698 (PMC8706688; doi:10.3390/molecules26247698)
Supplement: Supplementary file 1 [file molecules-26-07698-s001.zip › molecules-1483674-supplementary.pdf]

Supplementals

Table S1. Degradation ratio in percent of ascorbic acid (AsA), 5-caffeoylquinic acid (CQA), and quercetin-3-rutinoside (Rutin) with and without of iron, measured by HPLC. Values within ± 20 % are seen as stable.

ID	substance	Insert Concentration [mM]	Degradation ratio % 40 min without iron	Degradation ratio % 40 min with iron	Degradation ratio % 0 min without iron	Degradation ratio % 0 min with iron
1	AsA	0.3	26.76	94.93	stable	stable
2	CQA	0.3	stable	stable	stable	stable
3	Rutin	0.3	stable	stable	stable	stable
4	AsA	0.15	60.49	90.54	stable	26.30
	CQA	0.15	stable	23.71	stable	stable
5	AsA	0.15	65.47	89.38	stable	34.41
	Rutin	0.15	stable	32.63	stable	stable
6	CQA	0.15	stable	35.14	stable	stable
	Rutin	0.15	stable	stable	stable	stable
7	AsA	0.1	86.41	84.56	stable	48.58
	CQA	0.2	stable	36.92	stable	stable
8	AsA	0.1	81.93	84.33	stable	57.16
	Rutin	0.2	stable	23.49	stable	stable
9	CQA	0.1	stable	22.81	stable	25.47
	Rutin	0.2	stable	stable	stable	stable
10	AsA	0.2	44.08	92.32	stable	stable
	CQA	0.1	stable	37.81	stable	stable
11	AsA	0.2	51.67	92.02	stable	27.85
	Rutin	0.1	stable	39.56	stable	stable
12	CQA	0.2	stable	25.27	stable	stable
	Rutin	0.1	stable	stable	stable	stable
13	AsA	0.1	77.84	85.31	stable	52.79
	CQA	0.1	stable	58.06	stable	stable
	Rutin	0.1	stable	stable	stable	stable
14	AsA	0.075	87.44	85.65	20.16	63.85
	CQA	0.15	stable	49.16	stable	stable
	Rutin	0.075	stable	stable	stable	stable
15	AsA	0.075	84.79	79.77	stable	30.29
	CQA	0.075	stable	69.43	stable	stable
	Rutin	0.15	stable	stable	stable	stable
16	AsA	0.15	61.67	89.99	stable	39.51
	CQA	0.075	stable	56.69	stable	stable
	Rutin	0.075	stable	stable	stable	stable
17	AsA	0.06	90.05	75.97	43.98	34.77
	CQA	0.12	stable	68.83	stable	stable
	Rutin	0.12	stable	stable	stable	stable
18	AsA	0.12	72.82	87.21	stable	49.02
	CQA	0.06	stable	70.62	stable	stable
	Rutin	0.12	stable	stable	stable	stable

19	AsA	0.12	83.46	87.87	stable	50.25
	CQA	0.12	stable	55.96	stable	stable
	Rutin	0.06	stable	stable	stable	stable

* Degradation ratio [%] = [(insert concentration-concentration after 40 min of cooking)/insert concentration] *100
